# Venovenous Extracorporeal Membrane Oxygenation (VV-ECMO) Support in Anaplastic Lymphoma Kinase (ALK)-Positive Anaplastic Large Cell Lymphoma With Pulmonary Involvement at Presentation

**DOI:** 10.7759/cureus.113894

**Published:** 2026-08-03

**Authors:** Rui F Gomes, Marta M Sousa, Guilherme Sapinho, Daniel C Gomes, João M Ribeiro

**Affiliations:** 1 Department of Intensive Care Medicine and ECMO Referral Center, Unidade Local de Saúde Santa Maria, Lisbon, PRT; 2 Department of Intensive Care Medicine, Faculty of Medicine, Universidade de Lisboa, Lisbon, PRT; 3 Department of Hematology and Bone Marrow Transplant, Unidade Local de Saúde Santa Maria, Lisbon, PRT

**Keywords:** acute respiratory failure (arf), alk positive anaplastic large cell lymphoma, hematologic neoplasms, hematology-oncology, peripheral t cell lymphoma, pulmonary critical care, veno-venous extracorporeal membrane oxygenation (vv ecmo)

## Abstract

Anaplastic lymphoma kinase (ALK)-positive anaplastic large cell lymphoma is a rare and aggressive peripheral T-cell lymphoma typically diagnosed at advanced stages. Although venovenous extracorporeal membrane oxygenation (VV-ECMO) has been associated with poor outcomes in patients with active hematological malignancies, emerging evidence points to a potential benefit for carefully selected patients with severe acute respiratory failure due to cancer. We describe the clinical case of a 19-year-old male who initially presented with pleuritic chest pain and fever. Following a four-day hospitalization, he was discharged home with a diagnosis of acute pericarditis. Because of worsening symptoms with acute respiratory failure and newly developed lymphadenopathies, he was readmitted a few days later and diagnosed with nosocomial pneumonia. Despite treatment with broad-spectrum antibiotics, his condition worsened, necessitating mechanical ventilation and VV-ECMO to manage refractory hypoxemia. Subsequent investigation revealed the diagnosis of ALK-positive anaplastic large cell lymphoma with pulmonary involvement. Treatment with cyclophosphamide, doxorubicin, vincristine, etoposide, and prednisolone (CHOEP) chemotherapy led to significant clinical improvement, allowing successful VV-ECMO weaning. However, the disease relapsed shortly after with a comparable severity of respiratory failure, leading to a second VV-ECMO run combined with urgent BRESHAP (brentuximab vedotin, etoposide, methylprednisolone (Solu-Medrol), high-dose cytarabine (Ara-C), and cisplatin) chemotherapy. This strategy resulted in another successful VV-ECMO weaning. Although complete remission was achieved, stem cell transplantation was delayed due to pulmonary sequelae, and the patient declined further treatment. A second relapse occurred, and the patient died 12 months after diagnosis. This case report explores the potential utility of VV-ECMO as a bridge to recovery in carefully selected patients with treatable hematologic malignancies.

## Introduction

The overall prognosis of hematologic malignancies (HM) has improved significantly over the last few decades, reflecting advances in both diagnostics and therapeutics. However, long-term survival remains poor in several cases, particularly among patients with multiple myeloma and acute leukemia [1]. Historically, hospital mortality rates associated with critical illnesses resulting from HM and related complications have been notably high, ranging from 37.0% to 78.3% among ICU-admitted patients [2-4]. This has led to some reluctance to admit these patients to intensive care units (ICU) [5] and to support severe organ dysfunction, given the potential futility of critical care interventions [6].

Venovenous extracorporeal membrane oxygenation (VV-ECMO) is an advanced life-saving technique used to provide respiratory support to selected patients with severe acute respiratory failure (ARF) when invasive mechanical ventilation (IMV) is insufficient to maintain adequate oxygenation or carbon dioxide elimination, or when the risk of ventilator-induced lung injury is unacceptably high [7]. The use of this organ support technique for treating patients with HM and severe ARF has been the subject of extensive debate. Early studies have reported high mortality rates [8-10] and an increased risk of hemorrhagic and infectious complications [11]. Nevertheless, some studies have shown that VV-ECMO may be appropriate for selected patients with active malignancies, especially young patients with treatable cancers (hematologic or not) [11,12].

Anaplastic lymphoma kinase (ALK)-positive anaplastic large cell lymphoma (ALCL) is an uncommon type of peripheral T-cell lymphoma accounting for 1-3% of all non-Hodgkin lymphomas and approximately 15% of all T-cell lymphomas [13]. Its incidence is higher in the first decades of life (median age: 34 years) and shows a male predominance (male-to-female ratio of 1.5:1-2:1) [14]. It is considered an aggressive disease, usually diagnosed at advanced stages (III or IV), with extranodal involvement observed in approximately 60% of cases. The most frequently affected extranodal sites at presentation include the skin, bone, and soft tissues [13,15]. Lung involvement has been reported in a substantial proportion of pediatric patients but appears to be less common in adults [13,16].

ALK is a receptor tyrosine kinase belonging to the insulin receptor superfamily. Chromosomal translocations involving the ALK gene can result in the generation of oncogenic genes. The most common alteration is the t(2;5)(p23;q35) translocation, which generates the NPM-ALK fusion protein. This constitutively active kinase promotes tumorigenesis through phosphorylation and activation of multiple downstream oncogenic signaling pathways [13-15]. These genetic rearrangements have a significant impact on disease biology, activity, and epidemiology. ALK-positive ALCL affects younger patients, responds better to anthracycline-based chemotherapy, and carries a better prognosis than ALK-negative ALCL (five-year overall survival of 71% vs 15%). Notably, even in relapsed disease, some ALK-positive ALCL patients can be cured with intensive salvage therapy, which includes autologous stem cell transplantation [15].

## Case presentation

A previously healthy 19-year-old male was admitted to the Emergency Department of a district hospital with a five-day history of anterior pleuritic chest pain and fever. The initial work-up revealed normocytic normochromic anemia (Hb: 11.2 g/dL), leukocytosis (16,500/µL) with neutrophilia (12,500/µL), elevated C-reactive protein (CRP) (11 mg/dL), and mildly elevated troponin T (19 ng/L), as shown in Table 1. A 12-lead ECG showed ST-segment elevation (~1 mm) and PQ-segment depression in V4-V6 leads. Transthoracic echocardiography found a small posterior pericardial effusion and preserved biventricular systolic function without regional wall motion abnormalities.

**Table 1:** Results of laboratory tests obtained on the first and second emergency department admissions. Abbreviations: ED, emergency department; PaCO_2_, partial pressure of carbon dioxide in arterial blood; PaO_2_, partial pressure of oxygen in arterial blood; SaO_2_, arterial oxygen saturation Arterial blood gas analysis at the first emergency department admission was performed while the patient was breathing room air. At the second admission, arterial blood gas analysis was obtained while the patient was receiving supplemental oxygen at 4 L per minute through a nasal cannula.

Laboratory parameter	Reference range	First ED admission	Second ED admission
Hematology			
Hemoglobin	13.0-17.5 g/dL	11.2	9.8
Hematocrit	35.5-50.5 %	32.5	29.9
Mean corpuscular volume	80.0-99.0 fL	88.6	89.1
Mean corpuscular hemoglobin	27.0-33.5 pg	32.1	30.6
White blood cell count	4.0-11.0 × 10^9^/µL	16.5	20.0
Neutrophils	1.9-7.5 × 10^9^/µL	12.5	16.0
Eosinophils	0.0-0.5 × 10^9^/µL	0.14	0.17
Basophils	0.0-0.2 × 10^9^/µL	0.08	0.12
Linfocytes	1.0-4.8 × 10^9^/µL	2.52	1.39
Monocytes	0.1-1.0 × 10^9^/µL	1.04	2.29
Platelet count	150-450 × 10^9^/µL	216	298
Coagulation			
Prothrombin time	11.6 seg	11.2	12.0
Activated partial thromboplastin time	29.0 seg	28.4	31.1
Fibrinogen	200-400 mg/dL	526	587
Chemistry			
Urea	16-49 mg/dL	39	43
Creatinine	0.70-1.20 mg/dL	0.74	0.79
Sodium	135-145 mmol/L	137	141
Potassium	3.5-5.1 mmol/L	4.7	4.5
Chloride	98-107 mmol/l	100	104
Total bilirubin	<1.2 mg/dL	0.92	0.96
Aspartate aminotransferase	0-40 U/L	46	62
Alanine aminotransferase	0-41 U/L	27	28
Albumin	3.5-5.2 g/dL	3.2	3.0
Lactate dehydrogenase	100-250 U/L	332	341
Troponin T	<14 ng/L	19	23
C-reactive protein	<0.5 mg/dL	11.0	26.0
Procalcitonin	<0.5 ng/mL	2.14	2.96
Blood gas analysis			
pH	7.35-7.45	7.39	7.30
PaCO_2_	35-45 mmHg	42	53
PaO_2_	75-100 mmHg	82	68
SaO_2_	92.0-98.5%	95.6%	92.9%
HCO_3_^-^	22-26 mmol/L	24.5	25.2

The patient was diagnosed with acute pericarditis and admitted to the Internal Medicine Ward, where he was treated with colchicine and ibuprofen. A comprehensive clinical history was obtained, which was remarkable for unintentional weight loss over the previous three months. All microbiological cultures and serological tests for viral infections, autoimmune and systemic inflammatory diseases yielded negative results. Given his clinical improvement, the patient was discharged home on the fourth day of hospitalization, without a definitive etiological diagnosis, and with a scheduled follow-up consultation eight days after discharge.

**Figure 1:**
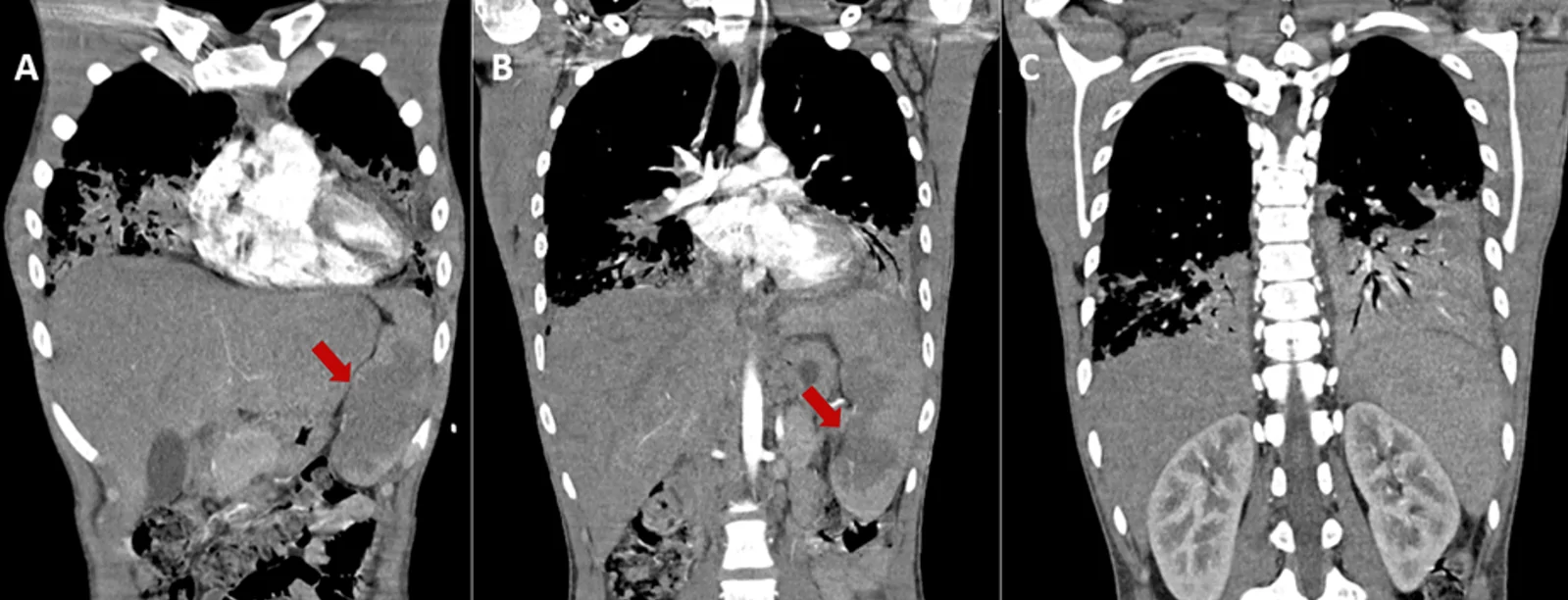
Coronal images of the contrast-enhanced chest, abdominal, and pelvic CT scan performed at the second hospital admission. Coronal images from the venous phase of the contrast-enhanced chest, abdominal, and pelvic CT scan obtained at the second hospital admission demonstrate bibasilar pulmonary consolidations with air bronchograms, predominantly involving the posterior segments of the lower lobes (image C). Hepatomegaly (image A) and splenomegaly (image B) are also identified. The spleen shows multiple hypodense lesions (red arrows), predominantly involving the perihilar and inferior aspects of the organ.

Five days later, he returned to the Emergency Department with relapsing fever, productive cough, and retroauricular, supraclavicular, axillary, and inguinal lymphadenopathies. On admission, he was placed on noninvasive mechanical ventilation (NIV) because of type 2 ARF (Table 1). Chest radiography was remarkable for the presence of bilateral opacities in both inferior lobes. Laboratory tests showed worsening leukocytosis (20,000/µL), neutrophilia (16,000/µL), and rising levels of inflammatory biomarkers (CRP: 26 mg/dL, procalcitonin: 2.96 ng/L). Contrast-enhanced chest, abdominal, and pelvic computed tomography (CT) revealed extensive consolidation in both inferior lung lobes, hepatosplenomegaly, and hypodense splenic lesions, as shown in Figure 1. No signs of pulmonary embolism were found.

A diagnosis of nosocomial pneumonia was formulated, and empiric antimicrobial therapy with piperacillin/tazobactam was started. The patient was admitted to the ICU, where progressive respiratory deterioration prompted endotracheal intubation and IMV on the fourth day of hospital stay (assist-control mode, tidal volume: 6.2 mL/kg of ideal body weight, respiratory rate: 24 breaths per minute, positive end-expiratory pressure (PEEP): 10 cmH_2_O, and FiO_2_ 100%). Distributive shock emerged after intubation, requiring high doses of norepinephrine (1.8 µg/kg/min), which was interpreted as septic shock. Refractory hypoxemia (PaO_2_/FiO_2_: < 90 mmHg) and nonprotective ventilation (plateau pressure: 28 cmH_2_O, driving pressure: 16-18 cmH_2_O) persisted, despite low tidal volume (4.8 mL/kg of ideal body weight), PEEP titration (PEEP: 12 cmH_2_O), permissive hypercapnia (PaCO_2_: 68 mmHg, pH: 7.26), neuromuscular blockade, and prone positioning.

Considering the extension of the lung lesions, their impact on respiratory mechanics, and the refractoriness of the ARF, the patient's condition was discussed with our ECMO referral center, and it was decided to cannulate on VV-ECMO as a bridge to recovery. On-site cannulation was performed, followed by transfer to our hospital. A concise timeline of the major clinical events is presented in Table 2.

**Table 2:** Chronology of major clinical events. Abbreviations: ARF, acute respiratory failure; CRP, C-reactive protein; CT, computed tomography; CXR, chest radiography; ECG, electrocardiogram; VV-ECMO, venovenous extracorporeal membrane oxygenation; ED, emergency department; FiO₂, fraction of inspired oxygen; Hb, hemoglobin; IBW, ideal body weight; ICU, intensive care unit; IMV, invasive mechanical ventilation; NIV, noninvasive ventilation; PEEP, positive end-expiratory pressure; RR, respiratory rate; TTE, transthoracic echocardiography; Vt, tidal volume Day 1 corresponds to the first hospital admission. Negative values indicate days before this first admission. Key clinical findings, laboratory and imaging results, possible diagnoses, and therapeutic interventions are shown for each timepoint.

Timepoints	Clinical events
Day -5 to Day 0	Anterior pleuritic chest pain, fever, and unintentional weight loss over the preceding 3 months.
Day 1 (first admission)	First ED admission: leukocytes of 16,500/µL (neutrophils: 12,500/µL), CRP of 11 mg/dL, troponin T of 19 ng/L. ECG: ST segment elevation ~1 mm and PQ depression in V4-V6. TTE: small posterior pericardial effusion, preserved biventricular function. Diagnosis of acute pericarditis. Started colchicine and ibuprofen. Admission to the Internal Medicine ward.
Days 1-4	Microbiological cultures, viral serologies, and autoimmune/systemic inflammatory work-up were all negative.
Day 4	Clinical improvement. Discharged without definitive etiological diagnosis, with follow-up consultation scheduled for 8 days later.
Day 9 (second admission)	Return to ED with relapsing fever, productive cough, and retroauricular, supraclavicular, axillary, and inguinal lymphadenopathy. Type 2 ARF → NIV. CXR: bilateral bibasilar opacities. Leukocytes 20,000/µL (neutrophils 16,000/µL), CRP 26 mg/dL, procalcitonin 2.96 ng/L. CT thorax/abdomen/pelvis: bilateral lower-lobe consolidation, hepatosplenomegaly, hypodense splenic lesions, no pulmonary embolism. Empiric piperacillin/tazobactam started. ICU admission.
Day 12 (ICU day 4)	Respiratory deterioration → intubation and IMV (assist-control, Vt 5.1 mL/kg IBW, RR 24/min, PEEP 10 cmH₂O, FiO₂ 100%). Post-intubation distributive shock requiring norepinephrine up to 1.8 µg/kg/min.
Day 12 onward	Refractory hypoxemia (PaO₂/FiO₂ < 90 mmHg) and nonprotective IMV (plateau 28 cmH₂O, driving pressure 16-18 cmH₂O) despite low tidal volume, PEEP titration, neuromuscular blockade, and prone positioning.
Days 12-13 (ICU Days 4-5)	Case discussed with ECMO referral centre → on-site VV-ECMO cannulation as bridge to recovery, followed by inter-hospital transfer.

Differential diagnosis

Despite some common features of bacterial pneumonia, such as fever, productive cough, leukocytosis, neutrophilia, increased inflammatory biomarkers, and new lobar consolidations on chest radiography and CT scan, the failure to improve with broad-spectrum antibiotics and the presence of incongruent clinical signs, such as unintentional weight loss, pericardial effusion, recurrent fever, generalized lymphadenopathies, and hepatosplenomegaly, prompted an extensive differential diagnosis. Table 3 shows all relevant results of the complementary diagnostic tests.

**Table 3:** Results of the relevant laboratory and microbiological tests. Abbreviations: ACE, angiotensin-converting enzyme; ALK, anaplastic lymphoma kinase; ANAs, antinuclear antibodies; ANCAs, antineutrophil cytoplasmic antibodies; Anti-CCP, anti-citrulline antibodies; BAL, bronchoalveolar lavage; BS, bronchial secretions; IGRA, interferon-gamma release assay; NFS, nasopharyngeal swab; RPR, rapid plasma reagin; RT-PCR, real-time polymerase chain reaction; TPHA, treponema pallidum hemagglutination

Laboratory test	Source(s)	Result
Biochemistry		
Urinary antigen (*Legionella pneumophila* and *Streptococcus pneumoniae*)	Urine	Negative
Angiotensin-converting enzyme (ACE)	Blood	34 U/L (normal range: 8-52 U/L)
Clinical microbiology		
Interferon-gamma release assay (IGRA)	Blood	Negative
Gram staining	Sputum, BS, BAL, blood, urine	Negative
Ziehl-Neelsen staining	Sputum, BS, BAL, blood	Negative
Cultures		
Blood cultures	Blood	Negative
Sputum cultures	Sputum	Negative
Bronchial secretions (BS) culture	BS	Negative
Bronchoalveolar lavage (BAL) culture	BAL	Negative
Urine culture	Urine	Negative
Molecular biopathology		
*Mycoplasma pneumoniae* RT-PCR	BS, BAL	Negative
*Chlamydophila pneumoniae* RT-PCR	BS, BAL	Negative
SARS-CoV-2 RT-PCR	NFS, BS, BAL	Negative
Influenza A and B virus RT-PCR	NFS, BS	Negative
Respiratory syncytial virus RT-PCR	NFS, BS	Negative
Adenovirus RT-PCR	NFS, BS	Negative
Rhinovirus RT-PCR	NFS, BS	Negative
*Pneumocystis jirovecii* RT-PCR	BAL	Negative
Cytomegalovirus RT-PCR	Blood	Negative
Epstein-Barr virus RT-PCR	Blood	Negative
Serology		
Galactomannan	Blood, BAL	Negative
Antinuclear antibodies (ANAs)	Blood	Negative
Antineutrophil cytoplasmic antibodies (ANCAs)	Blood	Negative
Anti-citrulline antibodies (anti-CCP)	Blood	Negative
Rapid plasma reagin (RPR)	Blood	Negative
Treponema pallidum hemagglutination (TPHA)	Blood	Negative

Negative interferon-gamma release assay, direct bacilloscopy, and nucleic acid amplification tests (NAATs) for *Mycobacterium tuberculosis* made the diagnosis of tuberculosis highly unlikely. Serological tests were also performed to exclude infections with *Chlamydophila pneumoniae *and *Mycoplasma pneumoniae*, which were negative. The same result was obtained for *Legionella pneumophila* urinary antigen testing.

The absence of mediastinal and/or hilar lymphadenopathies, parenchymal nodules, or ground-glass opacities on the chest CT scan was not suggestive of pulmonary sarcoidosis. The radiological findings were also not suggestive of viral pneumonia or pulmonary vasculitis. Not surprisingly, NAATs for common viral pathogens (SARS-CoV-2, influenza A and B, respiratory syncytial virus, adenovirus, and rhinovirus) and serological tests for antineutrophil cytoplasmic antibodies were negative. Bronchoalveolar lavage (BAL) was performed, yielding negative results for bacterial, fungal, and mycobacterial cultures, as well as NAATs for *Pneumocystis jirovecii* and *M. tuberculosis*.

In light of the growing suspicion of pulmonary involvement by a lymphoproliferative disease, a multidisciplinary meeting was held with the Hematology Department. As decided in this meeting, complementary investigations were performed, including bone marrow aspiration and biopsy, as well as an excisional biopsy of the left inguinal lymph node. Histopathological examination of these samples established a diagnosis of ALK-positive ALCL with small-cell pattern. BAL was subsequently performed to evaluate the suspected pulmonary involvement, but cytology and immunohistochemistry studies were inconclusive. Table 4 details the results of the histopathological exams.

**Table 4:** Results of the relevant histopathological exams. Abbreviations: ALK, anaplastic lymphoma kinase; CD, cluster of differentiation; EBV, epstein-barr virus; EMA, epithelial membrane antigen; Ki-67, antigen kiel 67

Histopathological exam	Result
Bone marrow aspiration (sternum bone)	Normocellular bone marrow, with an increased myeloid-to-erythroid ratio. Myeloid series shows a slight left shift in the maturation curve with 3% blasts, and hypergranularity in the granulocytic lineage. No foreign cells to hematopoiesis are observed.
Bone marrow biopsy (left posterior iliac crest)	Normocellular bone marrow with a slight left shift in the white cell maturation curve. Scattered large cells are observed with positive immunoreaction for CD30, ALK, and granzyme B.
Left inguinal lymph node biopsy	Presence of small-to-medium lymphoid cells with irregular nuclei, clear cytoplasm, numerous mitoses, high growth activity (Ki-67 ~70%), and significant blood vessel proliferation. These cells are immunoreactive for CD45, CD30, CD4, ALK, and granzyme B. Weaker expression of CD2, CD3, and EMA was found. Epstein-Barr virus (EBV) testing was negative.
Bronchoalveolar lavage cytology	Sediment composed of bronchial cells, numerous macrophages, and polymorphonuclear cells. Rare clusters of cells with large nuclei, prominent nucleoli, and scant cytoplasm. Inconclusive immunohistochemistry study.

Treatment

**Figure 2:**
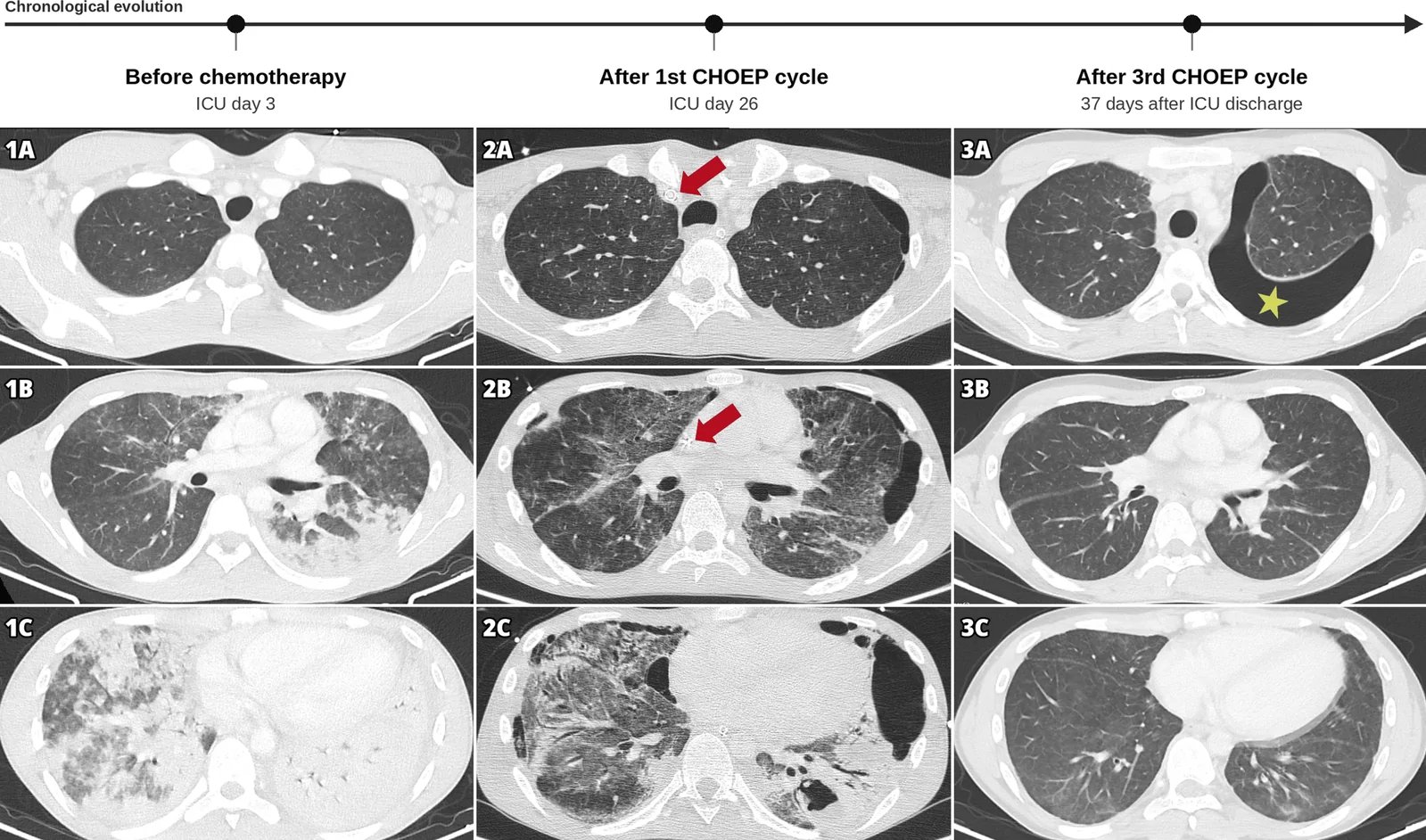
Evolution of the pulmonary lesions after starting CHOEP chemotherapy. Panels 1A-1C illustrate the lung lesions identified in the last chest CT scan performed before the initiation of chemotherapy. Panels 2A-2C correspond to the initial chest CT scan performed following the completion of the first chemotherapy cycle. Panels 3A-3C depict the progression of lung lesions after three chemotherapy cycles and successful VV-ECMO weaning. For each time point, the upper panels (A) show the lung apices, the middle panels (B) the mid-lung fields, and the lower panels (C) the inferior lobes. Extensive consolidation was present in both inferior lobes at baseline and showed progressive resolution with chemotherapy. Before completion of the first CHOEP cycle, the patient developed a bilateral pneumothorax that was still visible in panels 2A-2C. The partial resorption of this pneumothorax left a residual apical air collection (yellow star). As the patient was on VV-ECMO after the first CHOEP (cyclophosphamide, doxorubicin, vincristine, etoposide, and prednisolone) cycle, the return cannulas are visible in panels 2A and 2B (red arrows).

Intravenous methylprednisolone (1 g daily for four days) was started on day six after ICU admission due to the suspicion of pulmonary involvement by a lymphoproliferative disease. Following confirmation of the diagnosis, emergent cyclophosphamide, doxorubicin, vincristine, etoposide, and prednisolone (CHOEP) chemotherapy was initiated. Remarkably, a rapid improvement in bilateral lung consolidations was observed after the first chemotherapy cycle (Figure 2).

The patient continued on VV-ECMO until the completion of the second CHOEP cycle, culminating in successful VV-ECMO weaning after 38 days of ECMO run and ICU stay. The ECMO run was complicated by ICU-acquired weakness (ICU-AW), diaphragmatic dysfunction, heparin-induced thrombocytopenia, and membrane-associated coagulopathy, which prompted two oxygenator changes and resulted in a mild hemorrhagic event (tracheostomy bleeding). A small bilateral pneumothorax occurred during the ECMO run despite ultraprotective pressure-controlled ventilation.

Due to the severity of pulmonary lesions, ICU-AW, and diaphragmatic dysfunction, the patient experienced prolonged weaning from IMV. Percutaneous tracheostomy was performed to facilitate ventilator weaning. Following enhanced physical rehabilitation, the patient was transferred to the Hematology Ward (45 days after ICU admission) and completed the third CHOEP cycle without complications. A remarkable improvement in the lung lesions was documented on chest CT scan (Figure 2).

Outcomes and follow-up

The patient was discharged after 52 days of hospitalization with a plan to complete six cycles of CHOEP. Two months later, he was readmitted to the Emergency Department with severe ARF secondary to lymphoma relapse. Following a multidisciplinary consensus, the patient was readmitted to the ICU. Refractoriness to IMV led to VV-ECMO recannulation as a bridge to recovery under emergent second-line chemotherapy with BRESHAP regimen (brentuximab vedotin, etoposide, methylprednisolone, cytarabine, and cisplatin). Rapid clinical improvement enabled another successful VV-ECMO weaning without complications, after 11 days of extracorporeal respiratory support. Hospital discharge occurred on the 28th day after admission.

**Figure 3:**
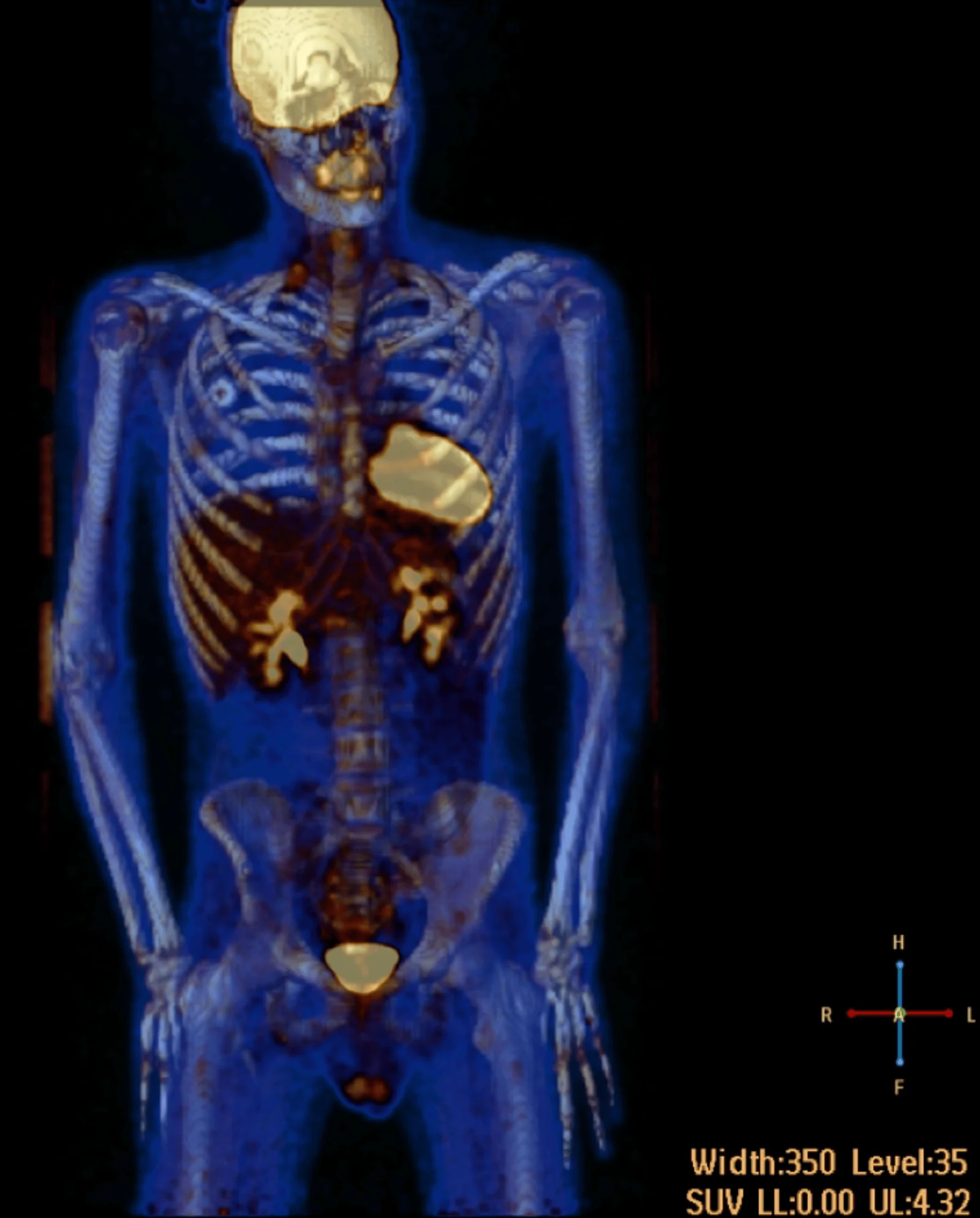
Three-dimensional reconstruction of the FDG PET/CT scan performed after the second BRESHAP cycle. A fluorodeoxyglucose (FDG) PET/CT scan was performed for lymphoma staging following the second cycle of BRESHAP (brentuximab vedotin, etoposide, methylprednisolone (Solu-Medrol), high-dose cytarabine (Ara-C), and cisplatin) chemotherapy. Physiologic FDG uptake was noted in the brain and myocardium, with normal tracer excretion via the renal collecting systems and urinary bladder (depicted in yellow on the color scale). No hypermetabolic foci suggestive of residual or active disease were identified. These findings are consistent with a complete metabolic response to the chemotherapy regimen.

After two BRESHAP (brentuximab vedotin, etoposide, methylprednisolone (Solu-Medrol), high-dose cytarabine (Ara-C), and cisplatin) cycles, complete remission was documented on PET/CT scan (Figure 3). Severe pulmonary sequelae precluded proceeding to hematopoietic stem cell transplantation, but a plan was made for two additional chemotherapy cycles while completing a pulmonary rehabilitation program. However, the patient declined further treatments. He remained stable for two additional months before experiencing another disease relapse, with clinical presentation and chest CT scan findings similar to prior episodes. This relapse was further complicated by severe diffuse alveolar hemorrhage (DAH).

The patient evolved with severe respiratory failure, which was refractory to noninvasive respiratory support. An extensive discussion was held with the patient regarding his expectations, perspectives on the disease, and preferences for further management. He expressed a strong desire to live and resume chemotherapy, regretting his previous decision to decline further treatments and fearing an early death. Nevertheless, he requested that invasive organ-support measures be limited to the minimum necessary to allow restarting chemotherapy. Based on this discussion, the patient was reintubated and readmitted to the ICU, but a multidisciplinary decision was made not to recannulate the patient for another VV-ECMO run, based on the perceived futility of this intervention, progressive clinical frailty, and in alignment with the patient's preferences.

The administration of high-dose steroids and emergent BRESHAP chemotherapy resulted in the resolution of DAH, allowing for extubation and the patient's transfer to the hematology ward, after 12 days of ICU stay. Following a short period of enhanced rehabilitation, he was discharged home on the 19th day of hospital stay, continuing an outpatient rehabilitation program and the hematologic treatments with crizotinib and ICE chemotherapy (ifosfamide, carboplatin, and etoposide). However, no clinical or metabolic remission was achieved. Subsequent PET/CT reassessment confirmed persistent metabolically active disease, consistent with refractory relapse. Due to disease progression, the patient died 12 months after the initial diagnosis.

## Discussion

ALK-positive ALCL often presents with systemic symptoms, including weight loss, fever, progressive weakness, fatigue, night sweats, and lymphadenopathies [15]. Extranodal involvement is frequently observed at presentation, with a higher prevalence in ALK-positive than in ALK-negative ALCL. The most affected extranodal sites include the skin, bones, liver, and soft tissues [14]. Bone marrow involvement is present in 10-20% of cases, and leukemic presentation can occur in patients with the small cell variant [14]. These characteristics make the diagnosis of ALK-positive ALCL challenging, as it requires consideration of several differential diagnoses.

The pulmonary involvement by ALCL can be primary (extremely uncommon) or secondary. Primary pulmonary ALCL is defined by clonal proliferation of lymphoid cells affecting one or both lungs without evidence of extrapulmonary disease within three months following diagnosis. Secondary pulmonary ALCL is diagnosed when clinical manifestations of systemic ALCL are present. The prevalence of pulmonary involvement in adults remains unknown, and the radiological presentation is nonspecific. Lymphomatous infiltration of the alveoli can lead to interlobular septal thickening and granulomatous consolidation on the chest CT scan, which may mimic pneumonia, miliary tuberculosis, or lymphangitic carcinomatosis [16]. In the case reported here, secondary pulmonary involvement was presumed on the basis of systemic ALK-positive ALCL accompanied by extensive bilateral pulmonary consolidations, which showed marked clinical and radiological improvement following the initiation of chemotherapy. Notably, this response occurred after a course of broad-spectrum antibiotic therapy that yielded no significant clinical improvement, and in the absence of any positive microbiological findings, rendering an infectious pneumonia unlikely.

ALK-positive ALCL exhibits a wide range of morphological features, further complicating definitive diagnosis [15]. Nevertheless, all cases are characterized by uniform CD30 expression, ALK gene rearrangements, and a variable proportion of hallmark cells with eccentric, horseshoe- or kidney-shaped nuclei with a prominent eosinophilic perinuclear clearing [15-17]. Most cases are identified by immunohistochemistry for aberrant ALK expression [18]. The differential diagnosis includes other ALK-positive neoplasms of non-T-cell lineage, such as ALK-positive large B-cell lymphoma, various carcinomas (including lung carcinoma), soft tissue neoplasms, and rare localized or systemic ALK-positive histiocytoses [17]. The risk of overlooking this diagnosis is not negligible and requires heightened levels of awareness.

Although ALK-positive ALCL carries a better prognosis than other peripheral T-cell lymphomas, involvement of more than one organ markedly worsens survival outcomes [19]. Additional factors associated with a poorer prognosis include older age, Ann Arbor stage III or IV, high International Prognostic Index (IPI) score, presence of bulky disease, low performance status, reduced serum albumin levels, high serum lactate dehydrogenase (LDH) levels, CD56 positivity, high serum soluble CD30 levels, and bone marrow involvement [15].

In the case of our patient, at least three factors associated with poor prognosis were identified at the time of presentation: involvement of multiple organs, Ann Arbor stage IV, and elevated LDH levels, which collectively translate into a high IPI score of three points. However, the definitive diagnosis was not yet established at the time of the first VV-ECMO cannulation, precluding consideration of these factors in the decision to initiate extracorporeal respiratory support. In contrast, the excellent response to the initial chemotherapy regimen, the patient's young age and good performance status, the potential for prolonged remission with intensive salvage therapy after relapse [15], and the better prognosis of lymphoma in the context of severe acute respiratory distress syndrome (ARDS) in cancer patients were decisive factors for the multidisciplinary decision to proceed with a second VV-ECMO run. At both time points, the absence of VV-ECMO cannulation would have significantly increased the likelihood of a fatal outcome.

A recent prospective, multinational observational cohort study (the YELENNA study) found no survival benefit associated with VV-ECMO support in patients with severe ARDS and cancer, of whom 73.4% had a hematological malignancy. Importantly, no deleterious effect was observed among those who received VV-ECMO, as the incidence of hemorrhagic and infectious complications was comparable between ECMO-supported and non-supported patients. Older age, peripheral vascular disease, severe ARDS at inclusion, acute kidney injury, and ICU admission as a time-limited trial were identified as independent predictors of higher 90-day mortality, whereas lymphoma was associated with lower 90-day mortality. On this basis, the authors advise caution when considering ECMO in patients with cancer and severe ARDS, while not excluding a possible benefit in selected subgroups, and therefore recommend an individualized approach [20]. In our view, such an approach should also rest on multidisciplinary discussion involving intensivists, hematologists, oncologists, and other relevant specialties, thereby allowing for adequate appraisal of the risks, potential benefits, specific treatment considerations, and expected outcomes.

To our knowledge, this is the first reported case of ALK-positive ALCL managed with VV-ECMO on two separate occasions, with successful weaning achieved on both. This report illustrates that ECMO is feasible and may serve as a bridge to recovery in patients with HM presenting with extensive but potentially reversible pulmonary involvement, provided that appropriate antineoplastic treatment is instituted. Nevertheless, no generalizations can be drawn from a single case report. The effectiveness and safety of this organ support technique, as well as its impact on medium- and long-term outcomes of patients with HM and severe ARF, remain insufficiently investigated and warrant further research.

## Conclusions

ALK-positive ALCL is an aggressive malignancy that is typically diagnosed at advanced stages. Its differential diagnosis is extensive and may be challenging. We presented a difficult-to-diagnose case of presumed secondary pulmonary involvement by ALK-positive ALCL with severe ARF requiring VV-ECMO support on two different occasions. The first cannulation was performed before the definitive diagnosis. The subsequent cannulation was based on a multidisciplinary decision that considered the patient's clinical status and several prognostic factors.

VV-ECMO support may be useful for the management of severe ARF in the context of HM. However, the benefit of this organ support technique on survival is not well established, warranting further investigation to clarify its efficacy in specific subpopulations. Reassuringly, no significant safety concerns have been mentioned in the literature, making this a possible option in carefully selected patients. This case report also underscores the importance of multidisciplinary management. Such an approach may facilitate the diagnosis of ALK-positive ALCL and other peripheral lymphomas, given its nonspecific clinical manifestations, and may improve the selection of the most appropriate management strategies by providing a broader perspective on disease severity, available treatment options, and anticipated outcomes.
